# DiSCount: computer vision for automated quantification of *Striga* seed germination

**DOI:** 10.1186/s13007-020-00602-8

**Published:** 2020-05-01

**Authors:** Raul Masteling, Lodewijk Voorhoeve, Joris IJsselmuiden, Francisco Dini-Andreote, Wietse de Boer, Jos M. Raaijmakers

**Affiliations:** 1 Department of Microbial Ecology, Netherlands Institute of Ecology (NIOO-KNAW), PO BOX 50, 6708 PB Wageningen, The Netherlands; 2 Institute of Biology, Leiden University, Leiden, The Netherlands; 3 Track32, Wageningen, The Netherlands; 4 School of Engineering and Applied Science, Rotterdam University of Applied Sciences, Rotterdam, The Netherlands; 5 Department of Plant Science, The Pennsylvania State University, University Park, State College, PA USA; 6 Huck Institutes of the Life Sciences, The Pennsylvania State University, University Park, State College, PA USA; 7 Chair Group Soil Biology, Wageningen University and Research (WUR), Wageningen, The Netherlands

**Keywords:** Machine learning, Deep learning, Computer vision, High-throughput assays, Parasitic weeds, *Striga hermonthica*

## Abstract

**Background:**

Plant parasitic weeds belonging to the genus *Striga* are a major threat for food production in Sub-Saharan Africa and Southeast Asia. The parasite’s life cycle starts with the induction of seed germination by host plant-derived signals, followed by parasite attachment, infection, outgrowth, flowering, reproduction, seed set and dispersal. Given the small seed size of the parasite (< 200 μm), quantification of the impact of new control measures that interfere with seed germination relies on manual, labour-intensive counting of seed batches under the microscope. Hence, there is a need for high-throughput assays that allow for large-scale screening of compounds or microorganisms that adversely affect *Striga* seed germination.

**Results:**

Here, we introduce DiSCount (**Di**gital **S**triga **Count**er): a computer vision tool for automated quantification of total and germinated *Striga* seed numbers in standard glass fibre filter assays. We developed the software using a machine learning approach trained with a dataset of 98 manually annotated images. Then, we validated and tested the model against a total dataset of 188 manually counted images. The results showed that DiSCount has an average error of 3.38 percentage points per image compared to the manually counted dataset. Most importantly, DiSCount achieves a 100 to 3000-fold speed increase in image analysis when compared to manual analysis, with an inference time of approximately 3 s per image on a single CPU and 0.1 s on a GPU.

**Conclusions:**

DiSCount is accurate and efficient in quantifying total and germinated *Striga* seeds in a standardized germination assay. This automated computer vision tool enables for high-throughput, large-scale screening of chemical compound libraries and biological control agents of this devastating parasitic weed. The complete software and manual are hosted at https://gitlab.com/lodewijk-track32/discount_paper and the archived version is available at Zenodo with the DOI 10.5281/zenodo.3627138. The dataset used for testing is available at Zenodo with the DOI 10.5281/zenodo.3403956.

## Background

The parasitic weed *Striga* is considered one of the major biotic constraints to food production in Africa, with crop yield losses reaching up to 100% [1]. The search for effective control strategies is urgent and subject of intense study, although no strategy to date is singularly effective. Current control strategies mainly focus on breeding for host resistance. Additionally, cultural methods such as hand weeding and a variety of soil management practices have also been used. Recently, there is a renewed search for specific chemicals of microbial [2, 3] and plant origin [4], but also synthetic compounds [5–7], that interfere at specific stages in the parasite’s life cycle.

One of the major targets of control strategies is the *Striga* seed bank that is widespread in millions of hectares of soil in Sub-Saharan Africa and Southeast Asia. Seed germination is a critical step in the life cycle of root parasitic weeds, which is induced by root exudate constituents that signal to the parasite that a host is nearby [8]. If *Striga* does not find a host after germination, the seed will decay after a few days, a process referred to as suicidal germination. On the other hand, when the parasitic weed can successfully attach to a host root via a haustorium, it will infect and syphon water and nutrients from the host. This leads to significant damage to the host plant even before the weed emerges from the soil. After emergence, the *Striga* plant grows and sets seed, leading to further accumulation of the parasitic weed’s seed bank, and consequently increasing the potential for new infections in the next growing seasons. Therefore, targeting control measures that interfere with the pre-attachment phases of the life cycle, i.e. germination and haustoria formation, holds great promise for controlling this root parasite.

A major bottleneck in discovering effective control measures and new candidate compounds is the labour intensity of working with the minute *Striga* spp. seeds (< 200 µm), particularly the manual evaluation of seed germination and the similar-sized soil particles and other impurities commonly found in seed batches. The assessment of germination rates using image analysis has been addressed for several plant species such as the model species *Arabidopsis thaliana* [9] and the non-model species *Helianthus annus* L. (sunflower) [10]. Building upon that, recent strategies and technical innovations are improving the throughput and accuracy of these tools. Technologies such as 3D-printed arrays coupled with image analysis were successfully used to assess the germination rates of Sainfoin, Amaranth (cultivar Liuye) and seasonal cabbage [11]. Here, we evaluated if such technologies could be developed for automated quantification of *Striga* seed germination. Due to the small seed size, simple staining methods to increase the contrast of *Striga hermonthica* seeds relative to the background [12] could facilitate computer vision. In order to automatically assess seed germination of root parasitic weeds, Pouvreau et al. [13] developed a high-throughput spectrophotometric method. Despite this recent development, parasitic weed seed germination assays are still mostly carried out manually using glass fibre filters [14–20]. The assays are done using a small filter (diameter ca. 1 cm) that contains ca. 50 to 100 *Striga* seeds, which allow for testing treatments with chemical compounds of interest. Further analysis is currently performed by manually counting total seed number and germination using a dissecting microscope.

Here, we introduce DiSCount (**Di**gital **S**triga **Count**er), a novel application of deep learning coupled with computer vision for automated quantification of *Striga* seeds and germination. The training input consisted of manually counted and annotated images collected in assays performed in the classic glass fibre filters. The current version of DiSCount quantitatively evaluates the images by discriminating seeds from soil particles and other debris, accurately inferring the total number of seeds in each image and the respective percentage of germination derived from the detection of the radicle onset. This process only takes ca. 3 s per image using a CPU, which represents ca. 100-fold speed increase when compared to manual seed counting, and ca. 0.1 s using a GPU, which represents ca. 3000-fold speed increase. By allowing rapid and reliable quantification of seed germination, DiSCount allows to substantially expand the number of chemical compounds as well as (micro) biologicals to be tested for their effects on *Striga* seed germination, either via stimulation (suicidal germination) or suppression of germination. Additionally, it facilitates the increase of the number of replicates, generating more statistically reliable results.

### Implementation

In this paper, object detection is used to localize and classify two types of objects: *Striga* seed and the seed radicle onset (germinated seed). Recent developments in object detection include Faster R-CNN with FPN [21], SSD [22], and YOLO [23–25]. The main trade-off between such models is speed versus accuracy, where a high localization accuracy, measured using the Intersection over Union score (IOU), is of lesser importance for this particular application. YOLOv3 performs well on Common Objects in Context (COCO) average precision (AP) 0.5 IOU benchmark with 57.9% mAP (mean Average Precision) compared to SSD (53.3% mAP) and Faster-RCNN (59.1% mAP) while using less computation time [25]. Considering the comparable performance of these networks, and the target system being standard lab PC’s that often lack CUDA capable GPU’s, YOLOv3 was selected as the most reasonable choice for broad public use. DiSCount is written in Python 3 with the PyTorch 1.2.0 library for deep learning [26], based on the code of Ayoosh Kathuria (https://github.com/ayooshkathuria/pytorch-yolo-v3). It makes use of the most recent version of the ‘You-Only-Look-Once’ object detector model, YOLOv3 [25] trained in Darknet [27], and several libraries, noted in the repository (installation manual). This object detector model is capable of localizing and classifying objects of varying sizes. It has also been proven to be useful in distinct tasks, such as self-driving cars [28] and nature conservation [29], mostly due to its localization and classification speed, accuracy and relative ease of implementation. The inference speed of the finished system, after deploying on a standard desktop Linux (Ubuntu 18.14.1 LTS with a 3.3 GHz Intel Core i5 processor and 8 GB of RAM) and Windows (Windows 10 64-bit with a 2.80 GHz Intel Core i7-6700T processor and 16 GB of RAM); is around 3 s per image. Using a system with a dedicated Graphics Processing Unit (GPU), an inference speed of 0.1 s per image was recorded (Windows 10 64-bit with 2.6 GHz Intel Core i7-9750HF, 16 GB of RAM and Nvidia GeForce RTX 2060 6 GB). The DiSCount software is available at 10.5281/zenodo.3627138 along with detailed training settings and a complete installation and user manual. The dataset used to test DiSCount is available at Zenodo, with the DOI 10.5281/zenodo.3403956.

#### Software workflow

**Fig. 1:**
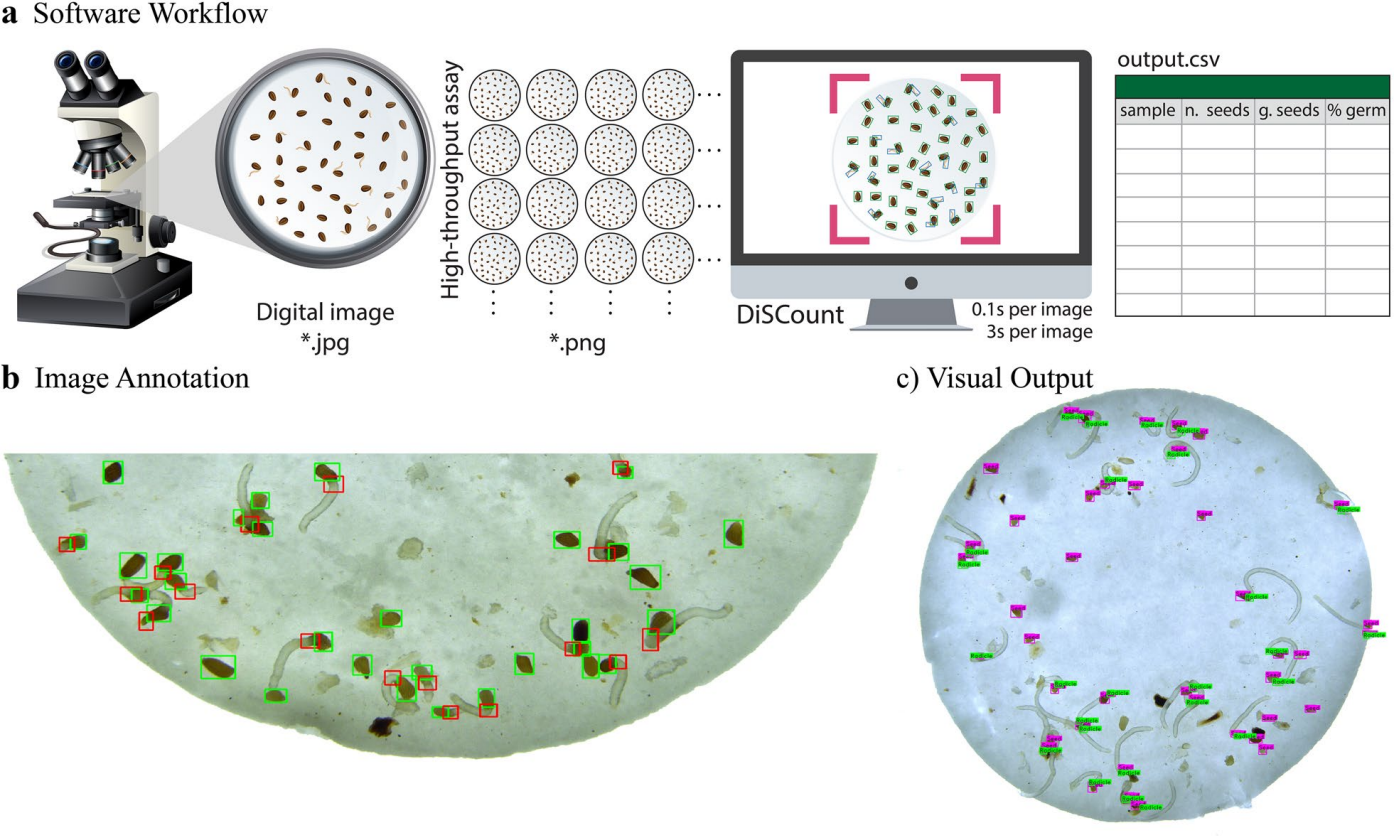
Overview of DiSCount. **a** The DiSCount workflow. After image acquisition and processing, DiSCount infers the number of germinated and total number of *Striga hermonthica* seeds in each image (ca. 3 or 0.1 s per image on a CPU and a GPU, respectively) and tabulates the obtained results in a *.csv file. **b** Example of annotated *Striga hermonthica* seeds (green bounding boxes) and their radicle onsets (red bounding boxes). **c** Example of DiSCount’s visual output. The visual output is a *.png file displaying seed objects (purple rectangles) and radicle objects (i.e., germinated seeds; bright green rectangles). The debris present from impurities in the seed batch are mostly not recognized by DiSCount, which indicates the potential use of DiSCount with less clean *Striga* seed batches

DiSCount consists of an object detector that estimates the total number of *Striga* seeds and radicles. An image (or set of images) is given as input file(s) and DiSCount provides two outputs, which include (i) a visual output of the analysed images in which the seed and radicle objects are distinctively identified, and (ii) a destination CSV file. The destination file (output.csv) is organized into four columns, containing the sample name (name of the input image), total number of seeds, number of radicle onsets (germinated seeds) and the percentage of germination (Fig. 1a).

#### Image acquisition and processing

After a 3 day exposure to microbial metabolites and synthetic chemical inducers of seed germination (rac-GR24, StrigoLab, Italy), each 13 mm Whatman GF/A glass microfiber filter with an average of 50 *Striga hermonthica* seeds (collected from the Abergelle agricultural field in Ethiopia) was individually imaged (.jpg format) using a dissecting microscope (Leica M205 C) with a camera attachment (Leica DFC450), with the lowest available magnification (i.e., 21.5 times). Due to this particular setup, the field of view when using the Leica Application Suite to record the images is smaller than the actual field of view in the microscope ocular, the result is that a full glass fibre filter does not fit in one image. Therefore, in this particular set-up, the images need to be merged for the full view of the filter assay before evaluation in DiSCount. Merging of the two images (top and bottom half of each filter) to make a full filter is done with “Photomerge” function in Photoshop (version 19.1.1) with Layout in “Auto” mode and “Blend Images Together” selected. Each image used for merging is of high quality with dimension of 2560 × 1920 pixels. The merged images (saved as *.png) have a dimension of circa 2500 × 2500 pixels. The correct functioning of DiSCount as an effective tool of seed germination assessment of *Striga hermonthica* requires the use of images with similar quality (Fig. 1c and Fig. 5).

#### The deep learning model

Developed by Redmon et al. [24], the ‘You-Only-Look-Once’ object detection network is a fully convolutional neural network, consisting of an image classifier followed by object detection layers. The image classifier is used as a feature extractor to produce feature maps. The resulting feature maps are then used to detect objects at different scales, enabling the detection of objects of different sizes. To assist the network in localizing objects during the training process, the network makes use of a grid of cells. Within each cell, three different anchor boxes are used for object detection [25]. Anchor boxes are pre-defined bounding-box shapes, which are used to base probable object shapes and locations on. To detect objects, the network takes an input image, rescales it to the appropriate size and produces an output matrix with four bounding-box coordinates, a confidence score and class probability scores. During the training stage, the outputs of the network are compared to ‘ground-truth’ annotations (i.e. manually counted images). The resulting error value (training loss) after comparison with the ground truth is then used to update the network weights (parameters) in order to improve predictions. This process requires to repeatedly run the training dataset through the network, in which each run of the dataset is one epoch.

#### Training

The network was pre-trained on ImageNet [30] for the feature extractor, after which the full object detection network was trained on 98 annotated images of *Striga hermonthica* seeds. The dataset was created by manually annotating two classes of bounding boxes; one for seeds and another for germinated seeds, by annotating the location of the radicle onset (Fig. 1b), using the LabelImg annotation software [31]. Images from different experiments and treatments were used in order to obtain a representative group of samples. Within the training dataset, a sample imbalance was observed of 2.5:1 for seeds and radicle onset, respectively. The training was performed on the Darknet framework [27] using adjusted anchor box dimensions that were derived from the training dataset by applying *K*-means clustering (using *K* = 9 clusters) to function with a 608 × 608 resolution (maximum standard setting of YOLO). Data augmentation was used, such as HSV (hue, saturation, value) values transformation, random horizontal flips and jittering, available in the Darknet environment [27]. The network was trained for 20,000 epochs using the Stochastic Gradient Descent optimizer, a momentum of 0.9, learning rate of 1*10^−3^ and a weight decay of 5*10^−4^. At epoch 16,000 and 18,000 the learning rate was divided by 10 to improve model fitting at those points (data not shown). The training settings can be derived from the configuration (.cfg) file in the repository, inside the config folder.

#### Model evaluation: validation and testing

**Fig. 2:**
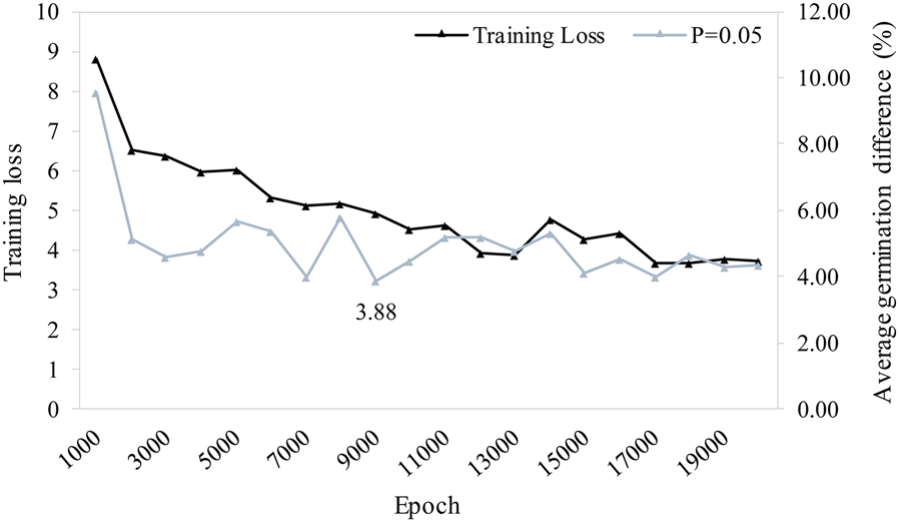
Training loss vs. average germination difference (%) of the network at threshold *P* = 0.05 over 20,000 training epochs. While the training loss continues to decline, the best local minimum was detected at epoch 9000 with a 3.88% difference on average in the germination rate per sample. The network at epoch 9000 was therefore selected and further validated to find the best threshold value of *P*

**Fig. 3:**
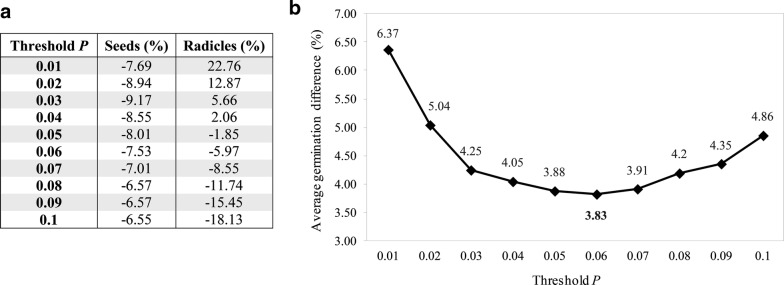
Variation of the threshold value *P* between 0.01 and 0.1. **a** Effect on the estimation of seed and radicle objects by varying the threshold P. **b** Average germination difference of the model at epoch 9000. A local minimum is found in the validation dataset at *P* = 0.06 with a 3.83% difference on the validation set, slightly better than the model at *P* = 0.05

The trained model was validated and tested using 188 manually counted images. A random selection of 94 out of these 188 images was used to validate the model and the remaining 94 to test the validated model. This reference (‘ground-truth’) contains the hand-counted number of seeds, radicles and respective germination percentage for each image, but not the actual location of each seed and radicle onset, differing therefore from the training data. The validation was performed at a resolution of 608 × 608 pixels for the input image and consists of finding the optimal settings (local minima) that the model can operate at in terms of the training epoch and the minimal object detection confidence threshold (also known as ‘objectness’), which determines what counts as a valid detection (below this threshold, a detection is discarded, effectively eliminating low-confidence detections). The aim was to determine the minimal difference in germination rate estimation over the validation set, i.e. a metric that reflects how accurate the model predicts the number of seeds and radicles within a given image. First, the validation of each model (training loss vs. the difference in germination rate estimation) was performed at the object detection confidence threshold of *P* = 0.05 (Fig. 2). This resulted in the selection of epoch 9000, since it has the lowest deviation from the ground-truth. Hereafter, this model was evaluated at different levels of object detection confidence by varying the threshold from *P* = 0.01 up to *P* = 0.1 (Fig. 3). From this evaluation, *P* = 0.06 was selected as best performing on the validation set, with an average difference in germination rate estimation of 3.83% (Fig. 3b).

## Results and discussion

After defining the optimal model parameters (model validation), the best performing neural network was then used to infer the germination percentages of the previously unseen testing dataset (i.e. the remaining 94 images) resulting in a test performance error of 3.38 percentage points averaged over the complete testing set. This result was obtained using absolute values that were compared to the ground-truth (data in GitLab repository).

**Fig. 4:**
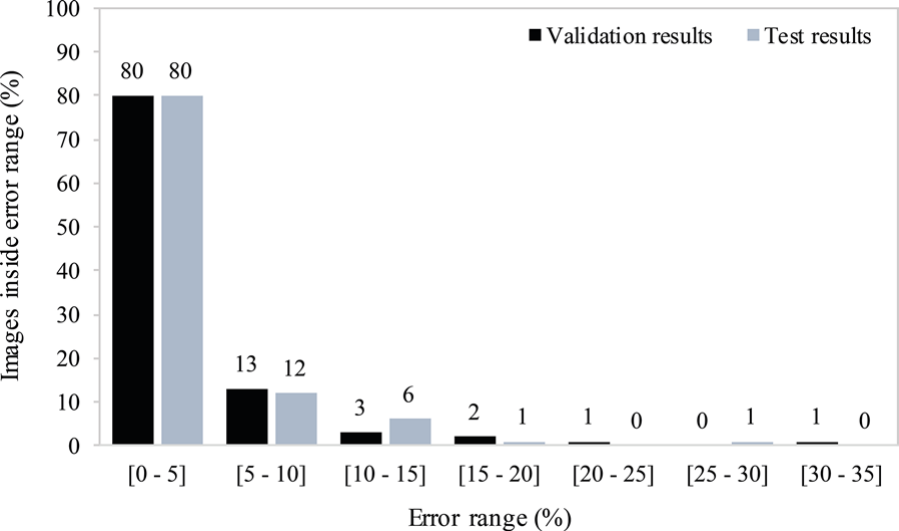
Frequency distribution of the accuracy of the estimated germination rate compared to the ground truth for each individual test image in the validation (back) and test (grey) datasets. The results show a similar distribution with 80% of the images in the 0–5% error range in both the validation and test data using the same neural network

Considering the testing dataset, the error of 80% of the images of the 94 images was within the 0–5% error range. The remaining error ranges of 5–10%, 10–15%, 15–20% and 25–30% contained 12, 6, 1 and 1 percent of the images, respectively (Fig. 4). The images belonging to the higher error range categories (15–20% and 25–30%) are generally images with a very high number of radicles (germinated seeds). When varying the object detection confidence threshold during validation, the underestimation of seeds was relatively stable (between − 6.55% and − 9.17%). However, the estimation of radicles varied between − 18.13% and 22.76% (Fig. 3a). It is likely that such variation is caused by sample imbalances observed in the training dataset (2.5:1).

**Fig. 5:**
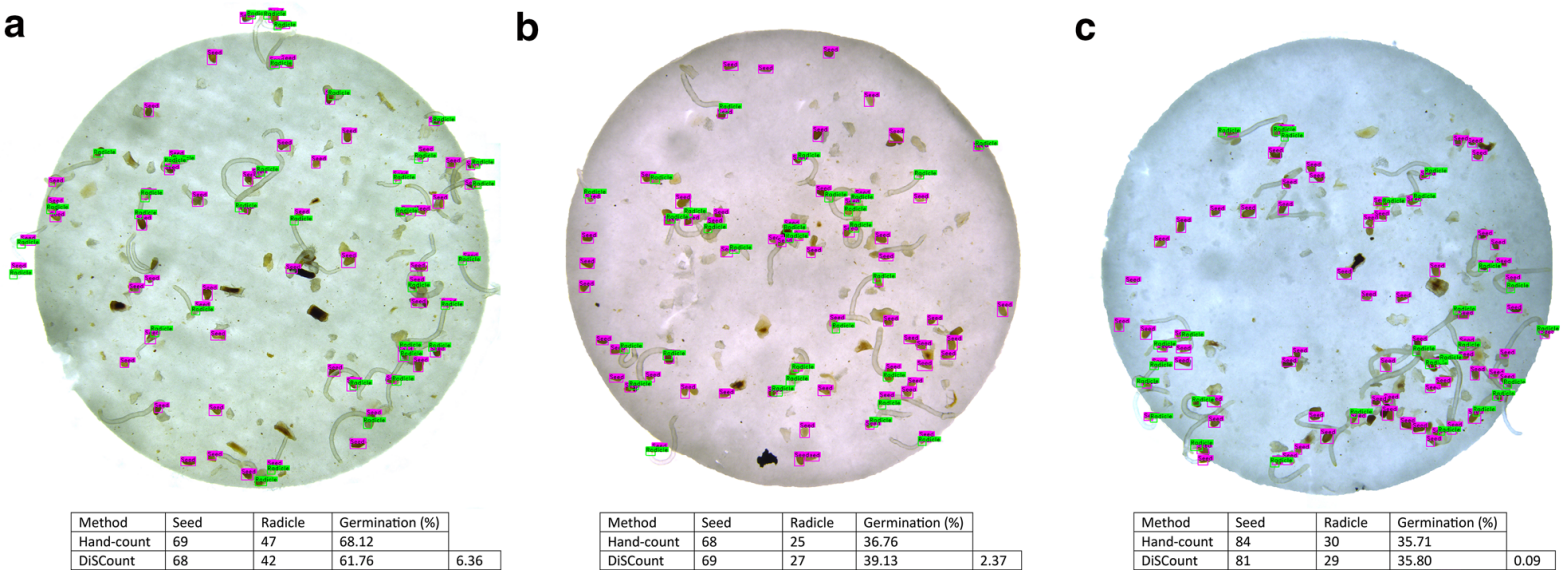
Example of output files from the testing dataset (**a**) Image 69-1, (**b**) Image 105-3, and (**c**) Image 63-3. The tables below each respective image indicate the comparison between Hand-count and DiSCount, with the respective germination and calculated differences

The most accurate model in inferring *Striga hermonthica* seed germination underestimates the number of seeds and radicle onsets (Table 1). In spite of this, it achieved a close match with respect to the difference in germination percentage (3.38 percentage points), despite the higher error rates of images with higher number of radicles. Due to the low average error rate and significantly faster inference time and low computational power required, DiSCount is a powerful tool to enhance experimental throughput in *Striga hermonthica* seed germination assays (Fig. 5).

**Table 1:** Test results of the model versus the hand-count

	Seeds	Radicles	Average germination (%)	Average difference per image (percentage points)
Hand-count	4848	910	21.42	3.38
DiSCount	4486	851	20.03	
Underestimation (%)	7.467	6.484		

An underestimate in seed and radicle onset count is balanced and results in a close approximation of the hand-counted results in terms of germination percentage. Data are available in discount_results.xlsx in GitHub repository

It is important to emphasize that class imbalance represents a possible factor accounting for high error rates. This factor particularly affects the object detection confidence of radicles (Fig. 3a). As such, a more balanced training dataset and retraining of the network could potentially improve the object detection confidence, resulting in an improved germination rate estimation. Despite DiSCount having a low threshold *P* (objectness) of 0.06 for object detection confidence, we found consistent results and successfully verified its performance with the test dataset. Nevertheless, it is possible that under more complex image analysis, for example on samples that contain more debris, or in case other developmental stages such as haustoria are included, these parameters need to be adjusted in order to increase performance of object detection. However, at this stage and with the specified experimental design and standardized quality of input images (standard lab protocol), DiSCount operates and performs in a stable manner providing reproducible results.

DiSCount can infer the total number of seeds and the number of germinated seeds without staining. Staining is usually a time-consuming procedure that aims at increasing contrast to facilitate visual inspection of the seeds [12]. For example, spectrophotometrically measuring the reduction of methylthiazolyldiphenyl-tetrazolium bromide (MTT) in 96 well-plates has enabled to rapidly quantify germination of root parasitic weeds [13], in order to assess the effect of several compounds and biological extracts on the germination of *Orobanche cumana*, *Orobanche minor*, *Phelipanche ramosa* and *Striga hermonthica.* Even though this assay also reduces the time needed to evaluate the phenotype of interest, DiSCount further reduces the time from the evaluation by reducing the experimental steps involved. This assay also is more appropriate to test soluble compounds, such as molecules of interest, plant root extracts and exudates, or extracts from microbial cultures. Apart from that, DiSCount also provides an interesting platform to test the effect of volatile compounds on seed germination.

The development of tools similar to DiSCount, using computer vision and machine learning, allows for the development of high-throughput screenings of different treatments (chemical or biological) against the destructive parasitic weed *Striga hermonthica*. Similar frameworks can be used to develop other computer vision systems with application in other fields, for example germination of spores of pathogenic fungi or hatching of nematode eggs. The most straightforward development to expand the application of DiSCount is the possibility to evaluate germination of other species of *Striga*, such as *S. asiatica* and *S. gesnerioides*, with relatively simple additional training. Possibly, also other plant parasitic weeds such as *Orobanche* species can be included. A significant improvement to DiSCount could be the recognition of an extra class, the haustorium, in addition to the seed and radicle. Automated recognition of haustoria development would expand the applicability of DiSCount, as it allows to screen for compounds or biological control agents that have an adverse impact on this essential structure in the parasite’s life-cycle.

**Fig. 6:**
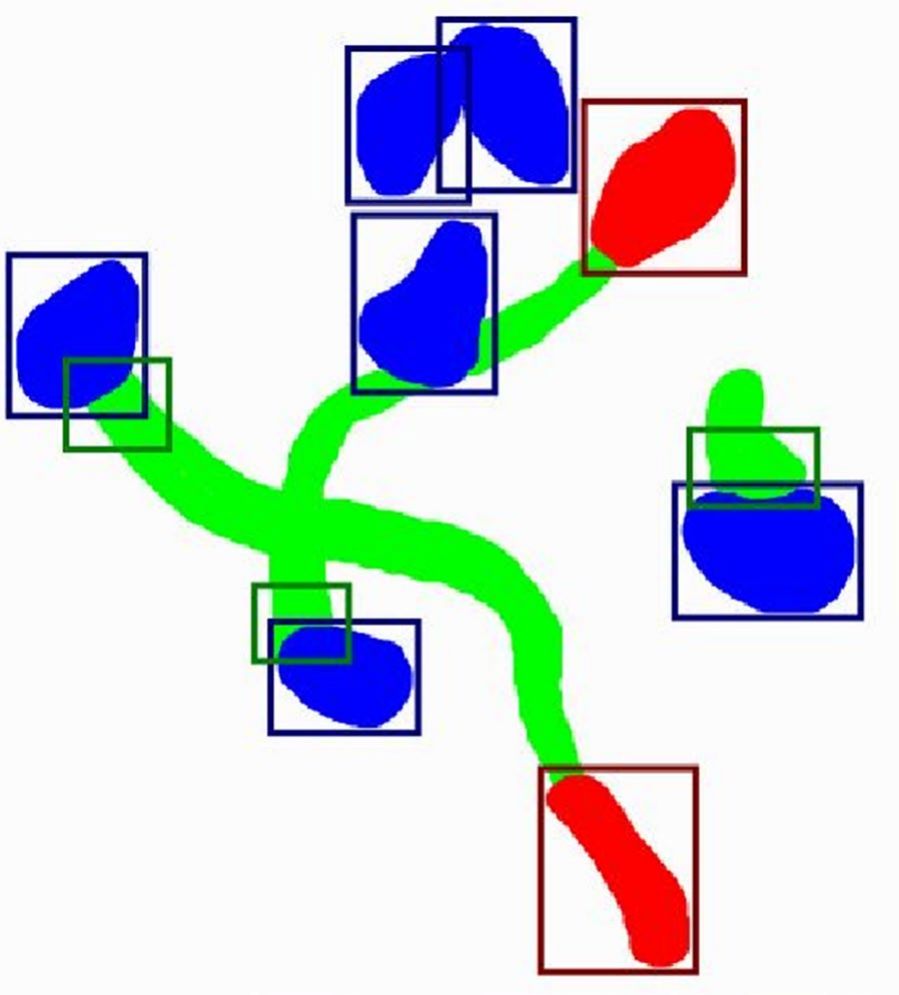
Example of the output to be achieved with an extended system. Bright blue: seed pixels, bright green: radicle pixels, bright red: haustorium pixels; dark blue bounding box: seed objects, dark green bounding box: radicle objects, dark red bounding box: haustorium objects. The input image contains 6 seeds, 3 of which are germinated and 2 that have developed haustoria. The extended version of DiSCount would likely compute a germination rate of 3/6 = 0.50 and haustoria development rate of 2/3 = 0.67. In this example, considering that the extended version detects 400 seed pixels, 300 radicle pixels and 100 haustoria pixels, the result would be mean individual object sizes of 400/6, 300/3 and 100/2, respectively. Considering that the camera characteristics are such that 1 pixel equals to 0.1 mm^2^, these would result in mean individual sizes of 6.67 mm^2^ (seeds), 10 mm^2^ (radicles) and 5 mm^2^ (haustoria)

Possible additional extensions include the measurement of total radicle and haustoria surface area in each image. These will allow to include a qualitative assessment of germination. This extension can be done using deep learning for semantic segmentation, enabling the counting of individual pixels belonging to each class and, as such, to estimate the mean individual sizes of each object (Fig. 6). Last, in order to facilitate research reproducibility and data management plans, a multi-user access user-friendly online solution can also be considered.

## Conclusions

DiSCount (**Di**gital **S**triga **Count**er) provides a high-throughput tool that accurately (average germination percentage difference of 3.38%) quantifies the germination of *Striga hermonthica* seeds using standard glass fibre filter as input images. Since germination is a critical stage of the life-cycle of parasitic weeds, discovering new control strategies that target seed germination is now a major focus. DiSCount reduces the time needed to assess the germination rates of *Striga hermonthica* seeds by at least 100 times (ca. 3 s per image) and up to 3000 times (ca. 0.1 s per image). Therefore, DiSCount has the potential to significantly accelerate the screening of compounds or microorganisms that interfere in the parasite’s life-cycle and help develop new control measures for this destructive parasitic weed.

## Data Availability

The software and user manual are freely available at https://gitlab.com/lodewijk-track32/discount_paper. The archived version of the repository is available at 10.5281/zenodo.3627138. The dataset analysed to assess the performance of the software is publically available at 10.5281/zenodo.3403956. The input dataset used to test the installation of the software is publically available at 10.5281/zenodo.3404131.
